# Current Pharmacological Strategies for Duchenne Muscular Dystrophy

**DOI:** 10.3389/fcell.2021.689533

**Published:** 2021-08-19

**Authors:** Shanshan Yao, Zihao Chen, Yuanyuan Yu, Ning Zhang, Hewen Jiang, Ge Zhang, Zongkang Zhang, Baoting Zhang

**Affiliations:** ^1^School of Chinese Medicine, Faculty of Medicine, The Chinese University of Hong Kong, Shatin, Hong Kong; ^2^Law Sau Fai Institute for Advancing Translational Medicine in Bone and Joint Diseases, School of Chinese Medicine, Hong Kong Baptist University, Kowloon, Hong Kong

**Keywords:** Duchenne muscular dystrophy, pharmacological therapeutics, skeletal muscle, fibrosis, inflammation, regeneration, meta-analysis

## Abstract

Duchenne muscular dystrophy (DMD) is a lethal, X-linked neuromuscular disorder caused by the absence of dystrophin protein, which is essential for muscle fiber integrity. Loss of dystrophin protein leads to recurrent myofiber damage, chronic inflammation, progressive fibrosis, and dysfunction of muscle stem cells. There is still no cure for DMD so far and the standard of care is principally limited to symptom relief through glucocorticoids treatments. Current therapeutic strategies could be divided into two lines. Dystrophin-targeted therapeutic strategies that aim at restoring the expression and/or function of dystrophin, including gene-based, cell-based and protein replacement therapies. The other line of therapeutic strategies aims to improve muscle function and quality by targeting the downstream pathological changes, including inflammation, fibrosis, and muscle atrophy. This review introduces the important developments in these two lines of strategies, especially those that have entered the clinical phase and/or have great potential for clinical translation. The rationale and efficacy of each agent in pre-clinical or clinical studies are presented. Furthermore, a meta-analysis of gene profiling in DMD patients has been performed to understand the molecular mechanisms of DMD.

## Introduction

As an X-linked neuromuscular disorder, Duchenne muscular dystrophy (DMD), is one of the most severe inherited muscle diseases and there is still no cure for DMD (Verhaart and Aartsma-Rus, 2019). The incidence of DMD is 1 per 5,136 male births (Crisafulli et al., 2020). The symptoms of DMD in infants, such as the slow motor development, will not be noticed until 3 years of age. Between 8 and 10 years of age, it is hard for patients to stand and walk, which may need the assistance of braces or wheelchairs. Cardiorespiratory failure is the leading cause of death in DMD patients (Meyers and Townsend, 2019; Silva et al., 2019). The life span of DMD patients is around 20 years, but the condition is fatal in 100% of cases (Chemello et al., 2020). DMD not only makes patients physically and mentally suffer tremendously but also imposes a heavy economic burden on their families and society (Gocheva et al., 2019).

Duchenne muscular dystrophy is caused by the mutations in the *DMD* gene (Monaco et al., 1986; Hoffman et al., 1987; Haidet et al., 2008; Okubo et al., 2020). The severity of DMD depends on the mutation type. ‘Out of frame’ mutations disrupt the reading frame and further produce the dysfunctional dystrophin, which result in the severe DMD phenotype (Aravind et al., 2019). On the other hand, ‘In-frame’ mutations perverse the frame of *DMD* gene and generate partial functional dystrophin protein, which lead to less severe Becker muscular dystrophy (BMD) phenotype (Haidet et al., 2008). In DMD, the absence of functional dystrophin and consequential loss of the dystrophin-associated protein complex (DAPC) increase the fragility of muscle fibers and lead to adverse cycles of muscle degeneration and regeneration (Ribeiro et al., 2019). In this condition, a continuous pathological environment including deteriorations of calcium homeostasis, activation of Ca^2+^-dependent proteases, inflammatory cytokines and mitochondrial dysfunction will further promote fibrosis and fatty tissue replacement (Klingler et al., 2012). To study the pathobiology of DMD, different animal models have been generated and studied (McGreevy et al., 2015). The most widely used animal model is mdx mice (Willmann et al., 2009), which carry a point mutation in exon 23 of the *Dmd* gene that results in the absence of full-length dystrophin (Sicinski et al., 1989). Mdx mice exhibit similar pathological features to DMD patients, including intense muscle fiber inflammation, muscle fibrosis and loss of muscle fibers (Stedman et al., 1991; Kupatt et al., 2021). However, two other proteins, utrophin and α7-integrin, fulfill the function of dystrophin in a compensatory way and minimize the dystrophic phenotype in mdx mice. Therefore, utrophin/dystrophin and integrin/dystrophin double-knockout mice have been generated, which have shown more similar pathological features with DMD patients (Guo et al., 2006; Rooney et al., 2006). Dystrophin-deficient dogs are also regarded as an excellent DMD model (Duan, 2011), which share remarkably similar clinical symptoms and histological lesions with DMD boys (Duan et al., 2015).

There are two lines of therapeutic strategies for DMD so far. One is dystrophin-targeted therapies that aim to restore the function of dystrophin. And the other one is to target the downstream pathological changes (Guiraud and Davies, 2017). Dystrophin-targeted therapies include gene therapies, cell therapies and protein replacement therapies. Despite massive pre-clinical and clinical studies have been carried on, there are challenges exist for the dystrophin-targeted therapies (Chamberlain and Chamberlain, 2017). The first concern is that the dystrophin-targeted therapies can just slow down the progression of DMD, but not restore the function of abnormal muscle tissues due to the degenerative nature of the DMD. Secondly, it is difficult for dystrophin-targeted therapies to target all muscle tissues, because muscle tissue is highly abundant and widely distributed throughout the whole body (Verhaart and Aartsma-Rus, 2019). Therefore, in addition to investigating strategies to cure dystrophin deficiency, increasing efforts are being placed on improving muscle function by targeting the downstream pathological changes of DMD. However, the curative effects of downstream specific therapies are not fully unity and the accurate mechanisms are obscure. It is desirable to have an in-depth understanding of the secondary pathological changes and develop more effective therapies.

This review introduces the current therapeutic strategies for DMD that have entered the clinical phase and/or have great potential for clinical translation. Furthermore, a meta-analysis of gene profiling in DMD patients is performed to understand the molecular mechanisms of DMD. The hub genes achieved from a meta-analysis may provide the potential targets for the treatment of DMD.

## Therapeutic Strategies Targeting Dystrophin

### Gene-Based Therapeutic Strategies

Gene-based therapeutic strategies have the potential to provide long-lasting and one-time treatment for DMD. In DMD, the deleted exons concentrate in the region from exon 43 to 55 (Asher et al., 2020), which make up roughly 60–65% of the events (Zimowski et al., 2014). Several gene-based therapeutic strategies including exon skipping, stop codon readthrough, gene-addition and gene-editing therapy have been designed.

#### Exon Skipping

Exon-skipping therapies target mutant codons with predesigned antisense oligonucleotides (AONs) to generate the proteins that can display partial function of dystrophin (Echevarría et al., 2018). AONs are used to control pre-mRNA splicing in specific sites and further obtain an in-frame message by removing one proximal exon to the deletion breakpoint (Kinali et al., 2009). In 2016, the US Food and Drug Administration (FDA) have approved the Eteplirsen (Exondys 51) that can specifically skip exon 51 in defective gene variants for DMD (Lim et al., 2017). Eteplirsen shows promising results for 6-min walk test (6MWT) that measures the distance a patient can independently walk within 6 min (Cirak et al., 2011). In 2019 and 2020, Golodirsen and Viltolarsen have been approved by the FDA, respectively (Heo, 2020; Al Musaimi et al., 2021). Both drugs are designed for patients with mutation that is amenable to exon 53 skipping (Dzierlega and Yokota, 2020). Forty to eighty week Golodirsen treatment significantly increases dystrophin protein expression in muscle of DMD patients (Frank et al., 2020). Similar beneficial result in dystrophin level is also observed in patients who received Viltolarsen treatment (Iftikhar et al., 2020).

However, the significant obstacle of exon-skipping therapies is that they can only provide treatment for patients with specific mutations. For example, skipping exon 51 therapy and skipping exon 53 therapy are applicable to around 14 and 8% of DMD patients, respectively (Echevarría et al., 2018). Besides, the long-term safety and efficacy of exon-skipping therapies are still needed to be tested. For example, Eteplirsen, as the first drug approved by FDA, its long-term efficacy has not been confirmed yet (Dzierlega and Yokota, 2020). Moreover, hypersensitivity reactions including upper respiratory tract infection, cough, and pyrexia have been reported in the latest clinical data of Viltolarsen and Golodirsen (Clemens et al., 2020; Heo, 2020).

Except for single exon skipping therapies, double exon skipping therapies have also been undertaken in clinical trials (which target two different exons). However, unlike *in vitro* studies for double exon skipping, the therapy transferring two AONs into myofibers shows reduced efficacy *in vivo* (Mitrpant et al., 2009; Niks and Aartsma-Rus, 2017). Therefore, more extensive studies are required.

#### Stop Codon Readthrough

Some nonsense mutations can result in premature stop codons (PSCs) and produce dysfunctional dystrophin (Ng et al., 2021). Around 13% of DMD patients are caused by these nonsense mutations (Aartsma-Rus et al., 2016). Unlike frameshift mutations, the PSCs do not disrupt the reading frame but prematurely terminate the translation during protein synthesis. Some small molecules have been designed to combine with these PSCs and stimulate stop codon readthrough (Malik et al., 2010; Aartsma-Rus et al., 2016). One representative is Gentamicin (Baradaran-Heravi et al., 2017). However, continual use of Gentamicin is prohibited due to the side effects including renal toxicity and ototoxicity (Vitiello et al., 2019). Two derivatives of Gentamicin, NB74 and NB84, show reduced cell toxicity and improved readthrough efficiency *in vitro* (Nudelman et al., 2010). Another aminoglycoside antibiotic Ataluren (NPC-14) has shown effective readthrough activities in pre-clinical studies (Guiraud and Davies, 2017) and could potentially provide treatment for around 11% of all DMD patients with a nonsense mutation (Namgoong and Bertoni, 2016). In 2016, Ataluren has been approved by the European Medicines Agency (EMA) for the treatment of DMD (Finkel et al., 2013; McDonald et al., 2017). However, a Phase 3 clinical trial reported that patients aged 7–16 after receiving Ataluren for 48 weeks did not show significant changes in 6MWD compared to placebo-treated patients (McDonald et al., 2017). Based on this, the drug failed to receive approval from the FDA (McDonald et al., 2017).

#### Gene Addition

Because DMD is caused by the mutations in the *DMD* gene. A highly appealing gene therapy is to replace the dysfunctional *DMD* gene with normal gene (Ricci et al., 2019). Adeno-associated virus (AAV) is a viral vector that can infect skeletal muscle with high efficiency (Zhang et al., 2020), which could be used as a delivery vector. However, the transgene capacity of AAV is limited and full-length dystrophin mRNA transcripts of the *DMD* gene are exceptionally large for AAV (Duan, 2018). To address this obstacle, the whole dystrophin gene is abbreviated to partially functional micro-dystrophin gene and packaged into AAV (Banks et al., 2010; Ramos et al., 2019). Numerous AAV serotypes have been explored in animal models (Wasala et al., 2019). Among several serotypes of AAV, serotypes 1, 6, 8, and 9 are outstanding candidates for DMD therapy as they exhibit a potent tropism for striated muscles (Inagaki et al., 2006; Leborgne et al., 2019). Recently, there are several micro-dystrophins AAV-based clinical trials ongoing (NCT03362502, NCT03375164, and NCT03368742; Table 1). The data from the 19 patients who received 1-year treatment in a Phase 1b trial (NCT03362502) showed that PF-06939926 treatment could induce continuous production of the partial functional dystrophin protein in muscle tissues and improvements in motor function (Belluscio et al., 2021). PF-06939926 has received orphan drug designation by both FDA and EMA and has entered Phase 3 trial (NCT04281485). Another micro-dystrophin transgene (rAAVrh74.MHCK7.micro-dystrophin) has been designed and possesses higher delivery efficacy than PF-06939926 (Potter et al., 2021). In mdx mice, after 3-month rAAVrh74.MHCK7.micro-dystrophin treatment, the enhancement in the specific force of diaphragm and tibialis anterior muscle has been observed (Potter et al., 2021).

**TABLE 1:** Status of the current therapeutic strategies targeting the primary defect of Duchenne muscular dystrophy (https://www.clinicaltrials.gov).

Therapeutic strategies	Mechanism of action	Chemistry	Drug route	Current stage
**Exon skipping**				
Golodirsen	Exon 53 skipping	Antisense oligonucleotides	Intravenous	Approved (FDA)
Eteplirsen	Exon 51 skipping	Antisense oligonucleotides	Intravenous	Approved (FDA)
Viltolarsen	Exon 53 skipping	Antisense oligonucleotides	Intravenous	Approved (FDA)
Casimersen	Exon 45 skipping	Phosphorodiamidate morpholino oligomer	Intravenous	Phase II/III
SRP-5051	Exon 51 skipping	Peptide-conjugated phosphorodiamidate morpholino oligomer	Intravenous	Phase II
DS-5141b	Exon 45 skipping	2 ENA antisense	Intravenous	Phase I/II
**Stop codon readthrough**				
Ataluren	Readthrough strategy of nonsense mutations	Small molecule	Oral	Approved (EMA), confirmatory Phase III,
NPC14(Arbekacin Sulfate)	Readthrough strategy of nonsense mutations	Small molecule	Intravenous	Phase II
**Gene addition**				
PF-06939926	AAV9 gene therapy	Recombinant adeno-associated virus and codon-optimized human micro-dystrophin	Intravenous	Phase III
rAAVrh74.MHCK7	AAV9 gene therapy	Recombinant adeno-associated virus and codon-optimized human micro-dystrophin	Intravenous	Phase I/II
SGT-001	AAV9 gene therapy	Recombinant adeno-associated virus and codon-optimized human micro-dystrophin	Intravenous	Phase I/II
**Genome editing**				
CRISPR-Cas9	Removes DNA encoding a specific target exon	AAVs- CRISPR-Cas9 system	−	Pre-clinical
**Protein replacement**				
C1100 (Ezutromid)	Upregulation of utrophin	Small molecule	Oral	Phase II
rAAVrh74.MCK.GALGT2	Upregulation of utrophin	Recombinant adeno-associated virus and GALGT2 gene	Intravenous	Phase I/II
**Myoblast transplantation**				
Donor-derived myoblasts	Fuse with host muscle fibers	Myoblasts grown	Intravenous	Phase I/II

#### Gene Editing

CRISPR (clustered regularly interspaced short palindromic repeats) Cas 9 (CRISPR associated protein) gene editing as a powerful tool to model or even to rectify genetic abnormalities has been used for correcting mutations in the *DMD* gene (Wang et al., 2017; Lek et al., 2020). CRISPR/Cas9 is an adaptive immune system in bacteria against invading bacteriophages and later developed as a tool for DNA editing (Jinek et al., 2012). The specific double-stranded DNA breaks can be induced by CRISPR/Cas9 technology in the genome and further activate DNA repair systems (Karimian et al., 2019). And the DNA repair systems will correct missense mutations by homology-directed repair-based germline editing or non-homologous end joining (NHEJ)-based postnatal editing (Long et al., 2014, 2016; Bengtsson et al., 2017; Zhang et al., 2017). The AAVs have been wildly used for the delivery of CRISPR/Cas9 *in vivo* (Samulski and Muzyczka, 2014). In mdx mice, the correction of exon 23 deletion by AAV8-CRISPR/Cas9 therapy can restore the expression of functional dystrophin protein in skeletal myofibers and significantly enhance the muscle force (Nelson et al., 2016). Besides, another study has shown that AAV6-CRISPR/Cas9 can correct both exon 52 and 53 deletions and significantly increase the force generation in mdx mice (Bengtsson et al., 2017). CRISPR/Cas9 gene editing delivered by AAV vectors can potentially bring clinical benefits to about 60% of DMD patients (Echigoya et al., 2015; Ousterout et al., 2015a, b; Taglia et al., 2015). However, the efficiency of CRISPR/Cas9-mediated genome editing is highly dependent on the dose of AAV (Min et al., 2019b). Inevitably, the excessive immune responses caused by exogenous AAVs and Cas9 might pose a challenge to their effective clinical application (Zhang et al., 2020). Further studies are still needed to understand the mechanism and frequency of off-target effects (Echigoya et al., 2015; Feng et al., 2015; Hollinger and Chamberlain, 2015). The related research is ongoing to generate more-specific Cas9 enzymes (Zheng et al., 2018).

### Cell-Based Therapeutic Strategies

Cell-based therapies aim to insert normal genomes into dystrophic muscles by transplanting normal muscle precursor cells (Sun et al., 2020). The ideal cell source can deliver a copy of the intact *DMD* gene and affect both limb muscles and heart (Ramos and Chamberlain, 2015). Different types of stem cells have been applied in DMD animal studies and clinical trials (Sun et al., 2020). The transplantation of satellite cells from muscle biopsies leads to the increased expression of dystrophin in mdx mice (Xu X. et al., 2015). Besides, CD133^+^ cells, the multipotent stem cells that are derived from normal human skeletal muscles, also have myogenic potential (Meng et al., 2018). In mdx mice, the transplantation of CD133^+^ cells can generate satellite cells and dystrophin-positive myofibers (Meng et al., 2014). Unfortunately, both satellite cells and CD133^+^ cells show no obvious functional improvement in DMD patients (Torrente et al., 2007; Xu X. et al., 2015). The engraftment of human-induced pluripotent stem cells (hiPSCs) holds great promise to correct mutations in DMD patients (Uchimura et al., 2017; Mondragon-Gonzalez and Perlingeiro, 2018). The hiPSCs are obtained from DMD patients and can be genetically corrected to restore normal dystrophin expression by CRISPR/Cas9 mediated gene editing (Hochheiser et al., 2018). Transplantation of genetically corrected hiPSCs can successfully restore the expression of dystrophin in mdx mice (Young et al., 2016). Although these cell-based therapies have shown apparent engraftment in mdx mice, their limited abilities for cell survival and migration after transplantation negatively influenced their clinical application (Sun et al., 2020). Therefore, more attempts are needed to improve the engraftment rates of stem cells (Hicks et al., 2018; Wu et al., 2018).

### Protein Replacement

Utrophin is a structural and functional autosomal paralog of dystrophin (Loro et al., 2020). Due to the similar structure and function, utrophin has been proposed as an alternate for dystrophin (Guiraud et al., 2019). In mdx mice, the upregulated expression of full-length utrophin can prevent the development of muscular dystrophy (Péladeau et al., 2018). Therefore, it is a promising strategy to upregulate utrophin levels in DMD patients. C1100 (Ezutromid) is a small molecule utrophin modulator to treat DMD (Tinsley et al., 2011). In mdx mice, the treatment of C1100 leads to better muscle physiology and increases overall muscle strength (Tinsley et al., 2011). However, a Phase 2 clinical trial (NCT02383511) indicated that C1100 did not provide any significant benefit for DMD patients and the development of C1100 therapy was discontinued (Ricotti et al., 2016). The GALGT2 is an enzyme that can generate the cytotoxic T-cell (CT) glycan to protect dystrophic skeletal myofibers from injury (Xu R. et al., 2015). In mdx mice, the overexpression of GALGT2 increases the expression of utrophin and can greatly strengthen the muscle membrane’s resistance to injury (Xu et al., 2009). rAAVrh74.MCK.GALGT2 is the surrogate gene therapy that using rhesus serotype 74 (rAAVrh74) to deliver human GALGT2 in the heart and skeletal muscle (Chicoine et al., 2014; Zygmunt et al., 2019). The phase1/2 trial of rAAVrh74.MCK.GALGT2 is ongoing (NCT03333590). Although utrophin exerts favorable effects as a compensate for dystrophin, it still differs from dystrophin in some functions. One of the most significant difference is that utrophin cannot prevent functional ischemia during muscle contraction (Lai et al., 2009; Li et al., 2010). Besides, dystrophin binds microtubules to form a rectilinear lattice beneath the sarcolemma, whereas utrophin cannot (Belanto et al., 2014). Therefore, the combination of utrophin-based therapies with other dystrophin-targeted treatments for DMD is well worth considering (Guiraud et al., 2019).

## Therapeutic Strategies Targeting the Secondary Downstream Pathological Mechanisms

The loss of dystrophin triggers multiple pathological pathways, including fibrosis, inflammation, loss of calcium homeostasis, oxidative stress, ischemia, and muscle atrophy (Birnkrant et al., 2018). In addition to the therapeutic strategies that aim to restore the functions of dystrophin, the agents targeting the downstream pathological changes caused by dystrophin deficiency attract more and more attentions (Table 2).

**TABLE 2:** Status of the current therapeutic strategies acting on downstream pathological mechanisms of Duchenne muscular dystrophy (https://www.clinicaltrials.gov).

Therapeutic strategies	Mechanism of action	Chemistry	Drug route	Current stage
**Anti-fibrotic**				
Pamrevlumab	Monoclonal anti-CTGF antibody	Antibody	Intravenous	Phase II
Losartan	Angiotensin II type 1 receptor blocker	Small molecule	Oral	Phase II
Halofuginone	Inhibitor of collagen a1 and MMP2	Small molecule	Oral	Phase I/II (Suspended)
Infliximab	Antibody to human TNF-α	Antibody	−	Pre-clinical
Suramin	Inhibits TGF-β	Small molecule	−	Pre-clinical
Imatinib Mesylate (Gleevec)	Inhibits TGF-β	Small molecule	−	Pre-clinical
**Anti-inflammatory**				
Corticosteroids (Prednisone, Prednisolone, and Deflazacort)	NF-κB inhibition	Small molecule	Oral	Phase III
Edasalonexent	NF-kB inhibition	Small molecule	Oral	Phase III
Vamorolone (VBP-15)	NF-kB inhibition	Small molecule	Oral	Phase II
Increlex (R)	Recombinant IGF-1	Fusion protein	Subcutaneous	Phase II
TAS-205	Hematopoietic prostaglandin D synthase inhibitor	Small molecule	Oral	Phase II a
Flavocoxid	NF-kB inhibition	Small molecule	Oral	Phase I
Givinostat	Histone deacetylase (HDAC) inhibitor	Small molecule	Oral	Phase I/II
Tamoxifen	Estrogen receptor modulator	Small molecule	Oral	Phase III
**Reduction of muscle damage**				
**Ca^2+^ dysregulation**				
Rimeporide	Sodium–hydrogen exchanger 1 inhibitor	Small molecule	Oral	Phase Ib
AT-300	Blocks mechanosensitive Ca^2+^ channels	Small molecule	−	Pre-clinical
Recombinant Mitsugumin 53	Facilitates membrane repair at sites of injury	Recombinant proteins	−	Pre-clinical
BGP-15	Hsp72 inducer	Small molecule	−	Pre-clinical
Streptomycin	Nonspecific Ca^2+^ channel blocker	Small molecule	−	Pre-clinical
**Oxidative stress**				
Coenzyme Q10	Electron acceptor for NADH and succinate dehydrogenase	Small molecule	Oral	Phase III
Idebenone	Antioxidant	Small molecule	Oral	Phase III
*N*-acetylcysteine	Endogenous antioxidant	Small molecule	−	Pre-clinical
**Muscle ischemia**				
Sildenafil	PDE5 inhibitor	Small molecule	Oral	Phase I/II
L-Arginine	Metabolic support	Recombinant proteins	Oral	Phase I
Tadalafil	PDE5 inhibitor	Small molecule	Oral	Phase III (Terminated)
**Muscle atrophy**				
GLPG0492	β2-Agonist, increases cAMP	Small molecule	Oral	Phase I
Urocortin	Increases cAMP	Ca^2+^-independent phospholipase A2 type β	−	Pre-clinical
rAAV1.CMV.huFollistatin344	Delivery of follistatin using adeno-associated virus	AAV1-Follistatin	Intramuscular	Phase I/II
**Bone homeostasis**				
Zoledronic acid	Inhibits bone resorption	Small molecule	Intravenous	Phase III
Alendronate (ALN)	Improves bone mineral density	Small molecule	−	Pre-clinical

### Therapeutic Strategies Targeting Fibrosis

Fibrosis is a prominent pathological change in DMD (Zhou and Lu, 2010). As a response to chronic tissue injury and long-term over-immunosuppression, fibrosis is a harmful and uncontrolled wound-healing process characterized by excessive accumulation of extracellular matrix (ECM) proteins (Wynn, 2007). Fibrosis can affect all tissues/organs and is the major cause of mortality in DMD patients (Wynn, 2008). A longitudinal study showed that endomysium fibrosis on the initial muscle biopsies correlated with poor muscle strength and age at loss of ambulation in DMD patients (Desguerre et al., 2009). Fibrogenic cytokines can activate tissue fibroblasts to produce ECM proteins (Suntar et al., 2020). Transforming growth factor-β (TGF-β), connective tissue growth factor (CTGF) and tumor necrosis factor-α (TNF-α) play significant roles in this process (Walker et al., 2019). TGF-β is a major mediator of the fibrotic response which can promote the build-up of ground substance as well as regulate the expression of catabolic enzymes and other mediators (Mázala et al., 2020). CTGF is one of the important downstream mediators of the effects of TGF-β on fibroblasts (Gonzalez and Brandan, 2019), which correlates with fibrotic activities (Sakai et al., 2017).

#### TGF-β

It has been proved that the expression patterns of TGF-β, as well as its receptors, are associated with dystrophin deficiency in DMD (Mázala et al., 2020). Type I (TβRI) and type II (TβRII) are two receptors of TGF-β. TGF-β can directly bind to the TβRII and then activate TβRI. TβRI subsequently phosphorylates downstream Smad proteins and activated Smad proteins further regulate the transcription of fibrotic genes (Kemaladewi et al., 2014). The TGF-β pathway could be an important therapeutic target to reduce excessive fibrotic tissue deposition in DMD (Mázala et al., 2020). It is known that angiotensin II independently or cooperatively works with TGF-β to induce fibrosis via the angiotensin type 1 receptor (AT1) in a multitude of tissues outside of the cardiovascular and renal systems (Acuña et al., 2014). Losartan is an AT1 receptor blocker that can attenuate TGF-β signaling (Lee et al., 2015). Losartan has been reported to significantly decrease fibrosis of the diaphragm and restore muscle strength of extensor digitorum longus muscle in mdx mice (Cohn et al., 2007). However, subsequent studies showed a minimal functional benefit of Losartan in mdx mice (Bish et al., 2011). A limited human clinical study showed that 1-year Losartan treatment significantly improved the cardiac function of DMD patients, but there was no impact on the fibrosis in skeletal muscle (Allen et al., 2013). Halofuginone (Halo) is another small molecule that can inhibit phosphorylation of TGF-β dependent Smad3 (Turgeman et al., 2008). Halo could decrease the established fibrosis (diaphragm and quadriceps), collagen I and III expression and smooth muscle actin content after 10-week treatment in mdx mice (Huebner et al., 2008). However, in a Phase 1/2 clinical trial, the patient experienced serious adverse reactions and died after receiving a high dose of Halo for 2 weeks (NCT02525302). The corresponding clinical trials were terminated. Suramin is a TGF-β inhibitor that has shown antifibrotic function in mdx mice (De Oliveira Moreira et al., 2013). The treatment of Suramin reduces the level of creatine kinase and attenuates fibrosis in skeletal muscles in mdx mice (De Oliveira Moreira et al., 2013, 2017). Besides, Suramin can protect muscles against exercise-induced damage in mdx mice (Taniguti et al., 2011). Imatinib mesylate (Gleevec^®^), an antineoplastic drug, also shows the ability to inhibit the pro-fibrogenic activation of TGF-β. In mdx mice, Imatinib mesylate significantly reduces fibrosis and improves hindlimb grip strength (Huang et al., 2009). However, in contrast with the results of B10-mdx, imatinib has not improved the fibrosis in the diaphragm of D2-mdx mice, and an unanticipated side effect of imatinib in heart has been reported (Ito et al., 2013). Therefore, more-specific inhibitors with less side effects are needed.

#### Connective Tissue Growth Factor

Connective tissue growth factor is a central mediator in the pathogenesis of fibrosis and plays important roles in the proliferation of fibroblasts, ECM production, vascular regeneration or other biological activities (Song et al., 2017). CTGF is widespread in endothelial, smooth muscle, fibroblast, and cartilage. TGF-β1 can significantly increase the expression level of CTGF in human fibroblasts (Ramazani et al., 2018). Moreover, CTGF promotes the activation of TGF-β signaling (Ramazani et al., 2018). In mdx mice, CTGF correlates with the number of necrotic-regenerative foci in dystrophic muscle (Morales et al., 2018). the mdx mice with hemizygous CTGF deletion (mdx^–^Ctgf^+/–^) shows better muscle strength and attenuated skeletal muscle impairment and fibrosis (Morales et al., 2013). It has been reported that raised expression levels of CTGF and TGF-β1 in the skeletal muscle are positively associated with the clinical severity of DMD (Song et al., 2017). These studies demonstrate that CTGF plays a central role in regulating fibrosis of DMD, which implied that CTGF could be a promising target for DMD treatment. CTGF is composed of four functionally conserved domains: insulin-like growth factor binding protein (IGFBP), von Willebrand factor type C repeat (VWC), thrombospondin type-1 repeat (TSP1 or TSR), and cysteine knot-containing domain (CT) (Chen et al., 2020). CTGF can activate TGF-β signaling by directly binding to TGF-β1 through the VWC domain (O’Leary et al., 2004). Besides, integrin αvβ3 can be activated by binding to the CT domain (Chen et al., 2020). The activated integrin signaling pathway can trigger the expression of CTGF and further promote the accumulation of ECM constituents (Riquelme-Guzmán et al., 2018). FG-3019 is a fully human monoclonal antibody that interferes with the action of CTGF by specifically targeting the VWC domain of CTGF. Pre-clinical studies suggest that FG-3019 can penetrate into tissues to reduce effective tissue levels of CTGF (Morales et al., 2013). A Phase 2 trial of FG-3019 in non-ambulatory subjects with DMD is ongoing (NCT01890265).

### Therapeutic Strategies Targeting Inflammation

In DMD, an increased level of Ca^2+^ leads to fiber necrosis and subsequently causes a severe inflammatory response (Allen et al., 2016). This process is mainly mediated by NF-kB pro-inflammatory pathway (Altamirano et al., 2012). The NF-κB pathway is regulated by TNF-α and interleukin 6 (IL-6), which are responsible for assembling the I-κB kinase (IKK) catalytic complex subunits (De Pasquale et al., 2012). Activation of NF-κB can induce the translocation of p50/p65 into the nucleus, and subsequent transcription of genes encoding cytokines and response factors involving in immune response (Baldwin, 2001).

Currently, the anti-inflammatory treatment for DMD is long-term dosing with glucocorticoids, including prednisone, prednisolone, deflazacort, and vamorolone, via inhibiting the NF-κB pathway (St-Pierre et al., 2004; Biggar et al., 2006). Glucocorticoids have been shown to prolong independent ambulation, improve pulmonary function, and delay the onset of cardiomyopathy in DMD patients (Hammers et al., 2020). However, there are also severe side effects for long-term glucocorticoid treatments, such as weight gain and hypertension (Matthews et al., 2016). Even though glucocorticoids share the same functional principles, there are still differences in the effectiveness and severity of side effects. For example, prednisone is superior to prednisolone and deflazacort in improving strength and pulmonary function (Gloss et al., 2016). And in terms of side effects, deflazacort can cause less weight gain than prednisone after 1-year treatment (Matthews et al., 2016). The glucocorticoid alternatives with fewer side effects are needed (Gloss et al., 2016). Vamorolone is an optimized glucocorticoid drug that shows consistent anti-inflammatory efficacy and reduced side effects compared to prednisolone (Sreetama et al., 2018). A Phase 1/2 study of Vamorolone in DMD patients showed an improved profile with reduced typical glucocorticoid-induced safety concerns after 2-week treatment (Hoffman et al., 2019).

Flavocoxid is another anti-inflammatory small molecule that can inhibit activities of COX and 5-LOX enzymes and further inhibit the NF-κB, TNF-α, and MAPKs pathways (Norwood et al., 2010; Vita et al., 2021). A Phase 1/2 study of flavocoxid indicated that the treatment was well tolerated and there was a significant reduction in serum IL-1β and TNF-α in the group of DMD boys (NCT01335295). However, the exploratory outcome measures failed to show significant effects for short-term treatment (Vita et al., 2021).

TNF-α is one of the pro-inflammatory cytokines that can regulate inflammatory response (Grounds and Torrisi, 2004). In mdx mice, proinflammatory cytokine TNF-α strongly contributes to necrosis in muscle fibers (de Senzi Moraes Pinto et al., 2013). Therefore, the anti-TNF-α therapies may be an effective treatment for DMD. Remicade (Infliximab) treatment can significantly increase skeletal muscle strength and decrease both cardiac and skeletal fibrosis by blocking the TNF-α signaling pathway in mdx mice (Grounds and Torrisi, 2004; Ermolova et al., 2014). However, the long-term TNF-α blockade treatment leads to deleterious changes in heart function (Ermolova et al., 2014).

Tamoxifen (TAM) is a selective estrogen receptor modulator that can either mimic or antagonize estrogens in a tissue-dependent manner (Dorchies et al., 2013). TAM can elevate the level of pro-inflammatory cytokines and growth factors involved in muscle regeneration and fibrosis, such as TGF-β, insulin-like growth factor 1 (IGF1) and osteopontin (Nagy et al., 2019). And then leads to an increased capacity of muscle-purified mitochondria to buffer cytosolic calcium (Nagy et al., 2019). It has been reported that TAM treatment can improve the body force and leg muscle force in mdx mice (Dorchies et al., 2013). Recently, a Phase 3 safety and efficacy trial of TAM in DMD is ongoing (NCT03354039). However, there is no result posted so far. The exact mode of action of TAM on dystrophic muscle is not clearly understood and some concerns about possible oncogenic side-effects in eventual life-long use have been raised (Vitiello et al., 2019).

Inhibition of histone deacetylase (HDAC) could lead to decreased expression of inflammatory genes in mdx mice (Nguyen-Tran et al., 2014). Givinostat (ITF2357) is an HDAC inhibitor that is currently being developed for the treatment of DMD (Bettica et al., 2016). Givinostat has shown several functional and morphological beneficial effects in mdx mice, such as increased cross-sectional area of myofibers, restored muscle force, decreased inflammatory infiltrate and prevention from fibrotic scars, which contribute to counteracting the muscle loss and the functional decline that are typically observed in mdx mice (Colussi et al., 2008). A Phase 2 clinical trial of Givinostat (NCT03373968) for DMD has been completed (Bettica et al., 2016). The trial showed that Givinostat treatment could increase the number of muscle fibers and suppress fibrosis. However, the study was not able to show improvement in functional tests and authors attributed this to the small sample size (Bettica et al., 2016). Recently, a Phase 3 trial of Givinostat is ongoing (NCT02851797).

Edasalonexent is a small molecule drug that can significantly inhibit p65-dependent inflammatory responses (Hammers et al., 2016). Long-term administration of Edasalonexent, or the related analog CAT-1041 with equivalent pharmacology, has been demonstrated several disease-modifying characteristics, including attenuated fatigue in skeletal muscle and increased muscle mass in mdx mice (Hammers et al., 2016). In addition, suppressed inflammation and fibrosis result in increased exercise endurance and improved diaphragm function in DMD animal models (Donovan et al., 2017). A Phase 3 study of Edasalonexent (NCT03703882) reported a significant down-regulation of NF-κB pathway-related gene expression in peripheral mononuclear cells after 2-week treatment. However, mild diarrhea and headache were the most common adverse events of Edasalonexent (Donovan et al., 2017).

Increlex is a recombinant human IGF-1 (rhIGF-1) that also offers potential treatments for DMD patients (Rutter et al., 2020). rhIGF-1 can reduce the inflammatory response and macrophage infiltration by down-regulating the expression of the NF-κB signal pathway in mdx mice (Ji et al., 2020). Systemic treatment with rhIGF-1 can improve muscle function and regeneration in mdx mice (Barton et al., 2002; Gregorevic et al., 2002; Shavlakadze et al., 2004; Schertzer et al., 2007). The data of a 6-month trial showed that rhIGF-1 improved linear growth but did not change motor function (Rutter et al., 2020).

Prostaglandin D2 (PGD2) is an inflammatory mediator and Hematopoietic prostaglandin D synthase (HPGDS) can promote the production of PGD2 (Ricciotti and FitzGerald, 2011). The overproduction of PGD2 by HPGDS can aggravate inflammation and exacerbate muscle tissue damage (Nakagawa et al., 2013). PGD2-mediated inflammation is involved in the pathology of DMD but the mechanism is not clarified yet (Nakagawa et al., 2013). The inhibition of PGD2 production via HPGDS inhibition may be a potential therapy for DMD. A Phase 1/2 study showed a favorable safety profile of TAS-205, HPGDS inhibitor, in DMD patients (Takeshita et al., 2018). However, a larger trial is required to ensure the effectiveness of TAS-205 (Komaki et al., 2020).

### Therapeutic Strategies Targeting Muscle Damage

#### Ca^2+^ Dysregulation

In DMD, the deficiency of functional dystrophin leads to membrane tears and Ca^2+^ leakage (Whitehead et al., 2006). High resting and active Ca^2+^ levels are observed in myofibers of DMD patients (Law et al., 2020). The excessive cytosolic Ca^2+^ can further aggravate dystrophic pathology by promoting Ca^2+^-dependent proteinase cleaving intracellular proteins (Townsend et al., 2007). Streptomycin can be used as a non-specific blocker of Ca^2+^ channels (Teichmann et al., 2008). Long-term Streptomycin treatment can alleviate limb muscle pathology, including reduced fibrosis, increased sarcolemmal stability and promoted muscle regeneration in mdx mice (Whitehead et al., 2006). However, there is no positive effect of streptomycin treatment on the diaphragm and heart muscle (Jørgensen et al., 2011). Another Ca^2+^ channel blocker, AT-300 (GsMTx4), which can specifically block mechanosensitive Ca^2+^ channels and improve muscle force production and decreases muscle degeneration in mdx model (Yeung et al., 2005; Bush et al., 2017). AT-300 has been granted orphan drug designation by the FDA. However, it still takes time to conduct pre-clinical testing before clinical application (Spinazzola and Kunkel, 2016).

Another approach is to repair the plasma membrane (Weisleder et al., 2012). Mitsugumin 53 (MG53) is an essential and muscle-specific member in cell membrane repairing, which plays a vital role in protecting skeletal and cardiac muscle cells from numerous types of acute injuries or chronic physiological stresses (Cai et al., 2009). Injection of recombinant human MG53 protein leads to reduced tissue damage in skeletal muscles of mdx mice (Weisleder et al., 2012).

The Na^+^-H^+^ exchanger 1 (NHE1/Rimeporide) is a transmembrane protein not only regulates the cellular volume of Na^+^ and H^+^ but also participates in the regulation of intracellular Ca^2+^ (Chen et al., 2004). There is an increase of intracellular Na^+^ concentration in the skeletal muscle of mdx mice and DMD patients. The intracellular Na^+^ overload in muscles is accompanied with muscle edema, which plays a role in muscle degeneration (Weber et al., 2012; Mijares et al., 2014). Rimeporide is an inhibitor of NHE-1 and shows anti-inflammatory and anti-fibrotic effects in mdx mice (Ghaleh et al., 2020). The positive effects on several pharmacodynamic biomarkers, including IGFBP1 and IGFBP6, have been reported in the Phase 1b study of Rimeporide (Gidaro et al., 2017; Previtali et al., 2020).

#### Oxidative Stress

Oxidative stress is one of the hallmarks of dystrophic muscles. Overload of Ca^2+^ enhances the production of reactive oxygen species (ROS) and results in the elevated oxidative stress (Allen et al., 2016). ROS further exacerbates the Ca^2+^ induced mitochondrial dysfunction and necrosis (Allen et al., 2016). Coenzyme Q10 (CoQ10) is one potential additional therapy targeting oxidative stress in DMD (Spurney et al., 2011). CoQ10 is an antioxidant that functions as an electron acceptor for respiratory complexes I (NADH) and II (SDH) in the electron transport pathway. Exogenous supplement of CoQ10 into mitochondria can increase the oxidative activity of NADH, and further provide metabolic support to muscles (Mizobuti et al., 2019). Besides, CoQ10 also helps to reduce the excessive oxidation radicals and intracellular Ca^2+^ concentration in DMD muscles (Mizobuti et al., 2019). Pre-clinical studies have demonstrated that CoQ10 treatment can preserve muscle strength by 42% compared to controls in mdx mice (Mizobuti et al., 2019). A Phase 3 clinical study reported that CoQ10 could prevent the loss of respiratory function in DMD patients. The favorable safety and tolerability profiles of CoQ10 make it a potential option to ameliorate the ROS in DMD (Salehi et al., 2017).

Another drug that targets ROS is Idebenone. As a synthetic short-chain benzoquinone, Idebenone can reduce ROS and improve mitochondrial function, therefore alleviate the muscle damage induced by dystrophin deficiency (Servais et al., 2020). Idebenone has cardioprotective effect and improves exercise performance of mdx mice (Buyse et al., 2009). A Phase 3 clinical trial (NCT01027884) indicated the potential disease-modifying effect of Idebenone on the progression of early functional cardiac and respiratory parameters in DMD. However, there has no significant improvement been observed in upper limb muscle strength (Buyse et al., 2015).

*N*-acetylcysteine (NAC) is an endogenous antioxidant (McKenna et al., 2006). Several pre-clinical studies have shown that NAC administration can reduce the dystrophic pathology in both skeletal and cardiac muscle in mdx mice (Whitehead et al., 2008; Fauconnier et al., 2010; Terrill et al., 2012; de Senzi Moraes Pinto et al., 2013), which implies that NAC could be a potential therapy for DMD boys. However, the treatment of NAC shows significant suppression on body weight gain and muscle weight in mdx mice (Pinniger et al., 2017). Therefore, these potential adverse effects should be given more considerations in the future clinical application of NAC.

#### Muscle Ischemia

Dystrophin deficiency leads to down-regulated expression and mislocalization of the source of nitric oxide (NO) in skeletal muscle (Brescia et al., 2020). NO is used to stimulate the production of cyclic guanosine monophosphate (cGMP), and the NO-cGMP signaling pathway is required to balance muscle oxygenation and prevent excessive vasoconstriction caused by exercising (Percival et al., 2011). In mdx mice, the suppression of the NO-cGMP signaling pathway promotes skeletal muscle weakness, vascular dysfunction and defective exercise performance (Asai et al., 2007; Garbincius and Michele, 2015; Hörster et al., 2015; Brescia et al., 2020). The inhibitors of phosphodiesterase 5 (PDE5) can suppress cGMP breakdown and further amplify the signal of NO-cGMP pathways (Dombernowsky et al., 2018). Two PDE5 inhibitors Tadalafil (Cialis^®^) and Sildenafil (Viagra^®^) can significantly reduce dystrophic pathology in mdx mice (Khairallah et al., 2008; Kobayashi et al., 2008; Percival et al., 2011, 2012). These studies indicate that PDE5 inhibitors can be potential therapeutic strategies for DMD, even though the molecular mechanisms remain unclear. However, a Phase 3 clinical trial demonstrated that Tadalafil failed to attenuate the decline in ambulatory ability in DMD patients (NCT01865084). And the related study for tadalafil is being terminated for lack of efficacy (Victor et al., 2017). The other inhibitor, Sildenafil, showed no significant improvement in cardiac function in a clinical trial (Leung et al., 2014). Besides, due to the higher number of side-effects, the study of Sildenafil has been terminated (Leung et al., 2014).

In DMD, loss of dystrophin also reduces the activity of Nitric oxide synthases (NO) (Dombernowsky et al., 2018). In normal muscle tissue, intramuscular L-arginine can convert to NO. However, DMD patients have elevated synthesis of asymmetric dimethylarginine (ADMA) and diminished synthesis of Homoarginine (hArg), which results in reduced bioavailability of NO (Hafner et al., 2016). Therefore, to elevate L-arginine level seems to be a promising strategy to stimulate mitochondrial function and reduce the oxidative stress of DMD. It has been proved that L-arginine can decrease inflammation and enhance muscle regeneration in mdx mice (Hnia et al., 2008). Furthermore, a Phase 3 study indicated that treatment with a combination of L-arginine and metformin slowed muscle function decline in DMD patients (Hafner et al., 2019).

#### Muscle Atrophy

The maintenance of skeletal muscle mass mainly relies on the regulation of muscle protein synthesis and degradation (Selathurai et al., 2019). β2-agonists have been found to initiate satellite cell proliferation and promote muscle protein production, as well as to increase lean body mass in muscle injury models (Lynch et al., 2000; Zeman et al., 2000; Harcourt et al., 2007). DT-200 is a β2-agonists originally investigated to treat sarcopenia and cachexia (Jones et al., 2010; Cozzoli et al., 2013). In mdx mice, DT-200 significantly reduces the muscle atrophy in the hindlimb and improves the running performance (Cozzoli et al., 2013). However, trials in DMD patients have not yet begun.

Urocortins (Ucns) are neuropeptides related to the hypothalamic corticotropin-releasing factor (CRF) (Manning et al., 2017). CRF_1_R and CRF_2_R are two receptors of Ucns and positively couple to G-proteins, which can activate adenylyl cyclase and promote cAMP formation (Kishimoto et al., 1995; Samuelsson et al., 2004). The activation of the CRF2R and downstream cAMP upregulation can prevent the degeneration of the diaphragm and attenuate the loss of its force (Reutenauer-Patte et al., 2012; Silveira et al., 2020). Further studies in mdx mice demonstrated that Ucns improved dystrophic muscle structure and function (Reutenauer-Patte et al., 2012; Burns et al., 2018), implying that Ucns might have great potential as the therapeutic target for DMD.

As a member of the TGF-β superfamily, myostatin is a muscle-specific secretory protein that negatively regulates muscle growth (Han and Mitch, 2011; Tang et al., 2020). In myogenic cells, myostatin inhibits the differentiation of myoblast and decreases the expression of transcriptional regulators for cell proliferation (Langley et al., 2002). Therefore, myostatin inhibition is a promising approach for treating DMD (Al-Zaidy et al., 2015). In mdx, the inactivation of myostatin resulted in a significant increase in muscle mass (Ran et al., 2020). Follistatin (FST) is a myostatin-binding protein that can enhance muscle formation by neutralizing inflammation induced by myostatin (Tang et al., 2020). The AAV-mediated gene delivery of FST in mdx animals showed reversed muscle pathology and improved strength (Mendell et al., 2015). Follistatin-344 is an isoform of FST, has the beneficial effects on muscle size and function (Haidet et al., 2008). Recently, a Phase 1/2 trial of rAAV1.CMV.huFollistatin344 in DMD patients is ongoing (NCT02354781).

### Bone Loss

In DMD, the combination of different causes, including long-term glucocorticoid treatment, decrease of physical activity, release of dystrophin-deficient induced cytokines, and insufficiency of vitamin D, will lead to osteoporosis (Buckner et al., 2015; Mah, 2016). In mdx mice, low bone mineral density and bone strength have been reported (Rufo et al., 2011). Furthermore, DMD patients show a trend of increased bone loss and bone fragility (Bianchi et al., 2003; Perera et al., 2016; Tian et al., 2016). Therefore, bone health management is an important part of lifelong care for patients with DMD. Pamidronate and Zoledronic acid are two bone metastases treatment drugs (Yang and Du, 2015). A small sample study indicated that Pamidronate or Zoledronic acid could stabilize or even increase the height of compressed vertebrae and alleviate back pain in prepubertal DMD patients (Hawker et al., 2005; Sbrocchi et al., 2012). Besides, in an uncontrolled study of 16 DMD boys, alendronate maintained the BMD Z-scores of the total body and lumbar spine (Hawker et al., 2005).

## Candidate Gene Targets Predicted by Meta-Analysis

In addition to dystrophin, numerous pathophysiological consequences of dystrophin deficiency also offer various downstream targets for DMD (Ziemba et al., 2021). However, not all the therapeutic strategies based on these targets are promising, and the concerns of efficacy and safety limit the clinical application of most strategies. To eliminate the unpromising targets, further understanding of the molecular mechanisms of these pathological changes is needed. Recently, computational approaches and network analysis have been extensively and successfully applied to find new gene–disease associations and identify novel therapeutic targets of diseases (Barabási et al., 2011; Nabirotchkin et al., 2020). A meta-analysis based on the publicly available differential gene expression profiles and literature-extracted expression regulation network of patients with DMD has been conducted in this review to identify the key regulators of these pathological changes.

A search for gene expression datasets publicly available on the National Center for Biotechnology Information (NCBI), Gene Expression Omnibus (GEO) and the European Bioinformatics Institute (EBI) Array Express (AE) was retrieved. Student’s *t*-tests and fold-change (FC) filtering were conducted to screen for the differentially expressed genes (DEGs) between DMD tissues and paired healthy tissues using the R software ‘limma’ package (Ritchie et al., 2015). The expression profiling information of each DEG was log^2^-transformed and then used to calculate the mean value (M) and standard deviation (SD) for control and DMD groups. In addition, the ‘meta’ package was used for performing a comprehensive meta-analysis of data aggregated from multiple sources (Balduzzi et al., 2019). The DEGs which satisfied the criterion of *P*-value < 0.05 and standardized mean difference (SMD) > 0.5 in the meta-analysis were identified as robust DEGs (Wang et al., 2019). Genes (STRING) database^1^ (version: 11.0) and Cytoscape software (version: 3.7.0) were utilized to construct protein–protein Interactions (PPI) networks on the robust DEGs (Olsen et al., 2014). A novel Cytoscape plugin cytoHubba (Chin et al., 2014) which could explore important nodes in biological networks was used for selecting hub genes by the cut-off value of Maximal Clique Centrality (MCC) score > 6 (Yu et al., 2020). Gene Ontology (GO) enrichment analysis of the robust DEGs was implemented with R package ‘cluster profiler’ and the Kyoto Encyclopedia of Genes and the Genomes (KEGG) was utilized on the robust DEGs (Yu et al., 2020).

**FIGURE 1:**
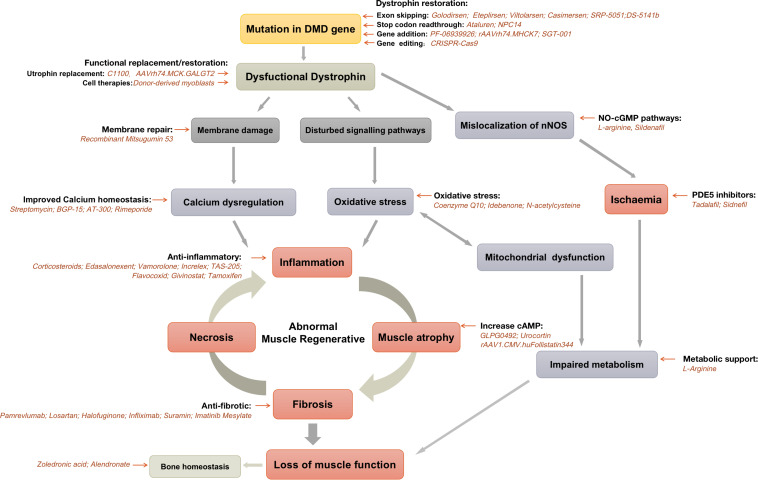
Current pharmacological strategies for Duchenne muscular dystrophy.

**FIGURE 2:**
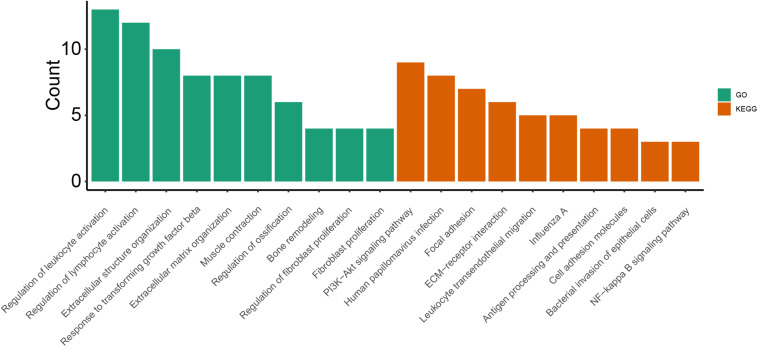
Enriched GO terms and KEGG pathways.

**FIGURE 3:**
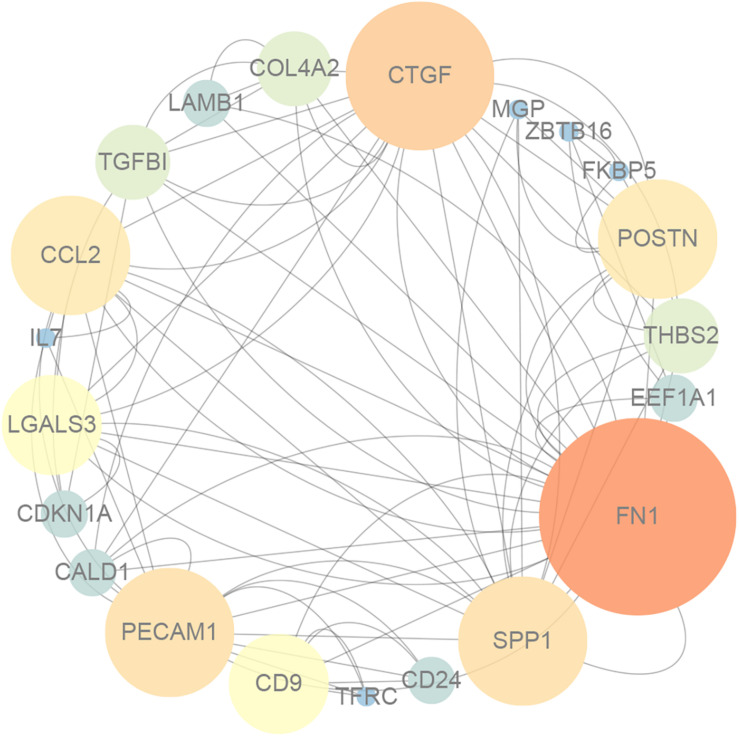
The protein–protein interaction network for DEGs, and the nodes with lighter color and bigger size represent the hub genes.

As shown in Figure 1, GO enrichment analysis results of DEGs indicated that changes in the cellular component of DEGs were mainly enriched in immune activation, ECM organization and regulation of fibrosis (Table 3). The KEGG signaling pathway analysis of DEGs showed that hub genes were mostly involved in the PI3K-Akt signaling pathway, human papillomavirus infection pathway and focal adhesion pathway. Moreover, 10 hub genes from the network were identified with the cut-off value of MCC score > 5, using the cytoHubba plugin of Cytoscape software. As shown in Table 4, Fibronectin 1 (FN1), CTGF, Secreted Phosphoprotein 1(SPP1), Periostin (POSTN), THBS2 (Thrombospondin 2), COL4A2, and TGF-β1 were involved in ECM formation. FN1 is an important component of ECM and serum FN1 level is a promising biomarker to monitor disease progression in DMD (Cynthia Martin et al., 2014). Both TGF-β1 and CTGF are the key regulators of fibrosis. Furthermore, as the upstream of TGF-β1, SPP1 can active TGF-β1 to induce collagen expression in skeletal muscle fibroblasts (Kramerova et al., 2019). COL4A2 and THBS2 also are two genes involved in the fibrotic process such as liver fibrosis and renal fibrosis (Ishikawa et al., 2015; Chen et al., 2019). However, further studies are needed to identify their roles in DMD. POSTN may participate in the decline of muscle necrosis in mdx mice (Ohlendieck and Swandulla, 2017). Except for the ECM-related genes, Galectin 3 (LGALS3), Platelet and Endothelial Cell Adhesion Molecule1 (PECAM1) and C-C Motif Chemokine Ligand 2 (CCL2) are mainly involved in the immune response. In the STRING database and Cytoscape software, robust DEGs were mapped into the PPI network and 89 interactions were selected by median confidence (more than 0.5) (Figure 2). The PPI networks are the networks of protein complexes formed as the results of biochemical or electrostatic forces. PPI network is crucial for molecular processes and abnormal PPI is the basis of many diseases. In this study, PPI network indicated that FN1, CTGF, PECAM1, POSTN, and CCL2 may play important roles in DMD (Figure 3).

**TABLE 3:** GO and KEGG pathways.

ID	Description	Gene	Function
GO:0002694	Regulation of leukocyte activation	CCL2/CD24/CD74/CDKN1A/HLA/DPA1/IL7/LGALS3/SFRP1/SOX11/TFRC/VAMP8/VSIG4/ZBTB16	Immune-related
GO:0051249	Regulation of lymphocyte activation	CCL2/CD24/CD74/CDKN1A/HLA/DPA1/IL7/LGALS3/SFRP1/SOX11/TFRC/VSIG4/ZBTB16	Immune-related
GO:0043062	Extracellular matrix organization	COL4A2/CTSS/FN1/LAMB1/LOXL1/PECAM1/PLTP/PXDN/SDCBP/SPP1	ECM-related
GO:0071559	Response to transforming growth factor beta	CLDN5/COL4A2/MXRA5/PENK/POSTN/SDCBP/SFRP1/SOX11	ECM-related
GO:0030198	Extracellular matrix organization	COL4A2/CTSS/FN1/LAMB1/LOXL1/PECAM1/PXDN/SPP1	ECM-related
GO:0006936	Muscle contraction	ADRA2A/CALD1/CASQ2/CHRNA1/MYBPH/MYL5/PGAM2/SGCA	Muscle development
GO:0030278	Regulation of ossification	CHSY1/MGP/SFRP1/SOX11/SRGN/ZBTB16	Bone development
GO:0046849	Bone remodeling	IL7/SFRP1/TFRC/TPP1	Bone development
GO:0048145	Fibroblast proliferation	CD74/CDKN1A/FN1/SFRP1	ECM-related
GO:0048144	Fibroblast proliferation	CD74/CDKN1A/FN1/SFRP1	ECM-related
hsa04151	PI3K-Akt signaling pathway	CDKN1A/COL4A2/ERBB4/FN1/IL7/LAMB1/SPP1/THBS2/THBS4	ECM-related
hsa05165	Human papillomavirus infection	CDKN1A/COL4A2/FN1/LAMB1/MX1/SPP1/THBS2/THBS4	Immune-related
hsa04510	Focal adhesion	CDKN1A/COL4A2/ERBB4/FN1/IL7/LAMB1/SPP1/THBS2/THBS4	ECM-related
hsa04512	ECM-receptor interaction	COL4A2/FN1/LAMB1/SPP1/THBS2/THBS4	ECM-related
hsa04670	Leukocyte transendothelial migration	CLDN5/MYL5/NCF2/NCF4/PECAM1	Immune-related
hsa05164	Influenza A	CCL2/HLA-DPA1/IFIH1/MX1/VDAC1	Immune-related
hsa04612	Antigen processing and presentation	CD74/CTSS/HLA-DPA1/PDIA3	Immune-related
hsa04514	Cell adhesion molecules	CLDN5/HLA-DPA1/PECAM1/PTPRF	ECM-related
hsa05100	Bacterial invasion of epithelial cells	ARPC1B/DNM1/FN1	Immune-related
hsa04064	NF-kappa B signaling pathway	BLNK/GADD45A/PLAU	Immune-related

**TABLE 4:** Hub genes selected by Maximal Clique Centrality (MCC) score.

Official symbol	Official full name	MCC score	Function	Category
FN1	Fibronectin 1	70	The encoded protein is involved in cell adhesion and migration processes including embryogenesis, wound healing, blood coagulation, host defense, and metastasis (Cai et al., 2018)	ECM-related
CTGF	Connective tissue growth factor	57	The encoded protein plays a role in different biological processes, such as cell proliferation, angiogenesis, and wound healing, as well as multiple pathologies, such as tumor development and tissue fibrosis (Chen et al., 2020).	ECM-related
SPP1	Secreted phosphoprotein 1	56	The encoded protein is involved in wound healing, bone homeostasis, and extracellular matrix (ECM) (Lamort et al., 2019).	ECM-related
CCL2	C-C Motif Chemokine Ligand 2	33	The encoded protein regulates the recruitment of osteoclast precursors to bone (Lorenzo, 2016).	Bone development
POSTN	Periostin	28	The encoded protein is a secreted extracellular matrix protein and involved in tissue development and regeneration, including wound healing, and ventricular remodeling following myocardial infarction (Hamilton, 2008).	ECM-related
LGALS3	Galectin 3	25	The encoded protein plays a role in numerous cellular functions including apoptosis, innate immunity, cell adhesion, and T-cell regulation (Hsu et al., 2009).	Immune-related
THBS2	Thrombospondin 2	24	The encoded protein plays a role in mediates cell-to-cell and cell-to-matrix interactions (Yang et al., 2000).	ECM-related
PECAM1	Platelet and Endothelial Cell Adhesion Molecule 1	13	The encoded protein is a member of the immunoglobulin superfamily and is likely involved in leukocyte migration, angiogenesis, and integrin activation (Woodfin et al., 2007).	Immune-related
COL4A2	Collagen Type IV Alpha 2	8	The encoded protein is the major structural component of basement membranes (Loscertales et al., 2016).	ECM-related
TGF-β1	Transforming Growth Factor Beta Induced	7	The encoded protein is an extracellular matrix protein and participates in many physiological processes including morphogenesis, adhesion/migration, angiogenesis, and inflammation (Ween et al., 2012).	ECM-related

The apparent enrichment of ECM-related genes suggests that most of the samples might be obtained from patients at an advantaged stage of disease progression. Indeed, the subjects might at different stages of DMD when the samples were obtained. Unfortunately, there is no information about the disease stage of the patients in each data set. The disease stages are largely associated with the age of patients since DMD is a progressive disease. We reviewed the clinical data of total 12 data sets and 7 of them (GSE1004, GSE1007, GSE6011, GSE13608, GSE38417, GSE43698, and GSE109178) showed the information of subject age. The mean age of subjects were calculated as following: GSE1004: 36.3 (months); GSE1007: 36.2 (months); GSE6011: 14.3 (months); GSE13608: 11 (months); GSE38417: 44 (months); GSE43698: 48 (months); GSE109178: 40.1 (months); All samples: 31.65 (months). The mean age of data sets GSE13608 is the lowest. The 10 top enriched pathways in GSE13608 are: hsa05416: Viral myocarditis; hsa05330: Allograft rejection; hsa05332: Graft-versus-host disease; hsa04940: Type I diabetes mellitus; hsa05320: Autoimmune thyroid disease; hsa04612: Antigen processing and presentation; hsa04514: Cell adhesion molecules; hsa04610: Complement and coagulation cascades; hsa05150: *Staphylococcus aureus* infection; hsa04145: Phagosome. The immune-related pathway (Antigen processing and presentation) and ECM-related pathway (Cell adhesion molecules) are the top enriched pathways in early age of DMD patients, implying that the ECM- and immune-related pathways have been involved in the pathological process in early stage of DMD.

## Discussion and Future Directions

Different therapeutic strategies for DMD have been developed. Numerous researches and clinical trials have been performed as detailed above. Nevertheless, there is still no effective disease-modifying therapy and many questions remain unsolved.

### The Challenges and Bottlenecks of the Therapeutic Strategies Targeting Dystrophin

Among the dystrophin-targeted therapeutic strategies, the therapies of exon skipping and readthrough blazed the new ways for DMD treatment (Ryan, 2014; Kole and Krieg, 2015). Three exon-skipping drugs, Golodirsen, Eteplirsen and Viltolarsen have received approvals from the FDA for DMD treatment and dozens more are being tested in clinical trials (Niks and Aartsma-Rus, 2017). Readthrough-based drug Ataluren has been marketed in some European countries for the treatment of DMD patients with nonsense mutations (Ryan, 2014). However, the most prominent problem is that exon skipping and readthrough therapies only available for DMD patients with specific mutation types. Therefore, more efforts are needed to improve the applicability of both therapies. Further innovations have been made in the field, such as multi-exons skipping therapies potentially benefit up to 45% of DMD patients. However, the efficacy of multi-exons skipping therapy is still controversial (Dzierlega and Yokota, 2020).

Gene-addition and gene-editing therapies are mainly restricted by the limitations of delivery with AAV vectors. Currently, AAV vectors are the most promising gene transfer tools due to their outstanding ability to target muscle (Wang et al., 2017). Unfortunately, AAV vectors have a limited carrying capacity and cannot deliver full-length *DMD* gene (Duan, 2018). One potential solution is to optimize AVV-based micro-dystrophin delivery therapies, which aim to restore the partial function of dystrophin. Another challenge for AAV-based therapies is an immunological risk, because high levels of AAV can lead to toxicity (Duan, 2018). The AAV vector with less immunotoxicity has been developed. AAVrh74 as a delivery vehicle has been found to be safe and tolerable in human studies, with minimal immune response (Mendell et al., 2009). Besides, the rAAVrh74.MHCK7.micro-dystrophin treatment has shown relatively low-risk adverse events in clinical studies (Mendell et al., 2020). However, some side effects, such as loss of appetite and vomiting, have still been observed in DMD patients (Nelson et al., 2017). And for gene-editing therapies, the effects of potential off-target events need to be investigated rigorously to ensure the long-term safety (Bengtsson et al., 2017; Min et al., 2019a).

Stem cells bring the promise for cell-based therapies due to their advantageous regeneration capability. Especially, the hiPSCs-based therapies, which can genetically correct and express functional dystrophin, hold the keys to treat DMD (Sun et al., 2020). However, many technical challenges and safety concerns still limit the clinical applications of cell-based therapies. Cultured satellite cells show limited transplantation efficiency and most satellite cells die after transplantation. Therefore, the delivery route needs to be optimized to obtain a higher muscle engraftment rate *in vivo*. Besides, the off-target effect of the gene-editing tools that used for hiPSCs construction, such as CRISPR/Cas9, is a big concern. Progress on overcoming these limitations is being made, including the identification of specific cell surface markers to separate the myogenic cells from the other types of cells, as well as the use of new generation CRISPR-based systems (Magli et al., 2017; Hicks et al., 2018).

To sum up, the restoration of dystrophin and the DAPC in DMD could prevent further muscle damage and slow disease progression theoretically. However, challenges such as tailoring to specific DMD mutations and achieving widespread delivery still need a long time to overcome.

### It Is Necessary to Identify More Effective and Safer Targets for the Treatment of DMD

Besides the dystrophin-targeted therapeutic strategies, great efforts have also been made to target the downstream pathogenic changes of dystrophin deficiency. The major advantage of these pharmacological strategies is that they are applicable to all DMD patients irrespective of their mutation type. Furthermore, most therapies introduced above have already been approved or under clinical studies for other diseases, which facilitates their clinical applications for DMD. Compared with gene-targeted and cell-based therapies, these molecular drugs with histories of clinical application show less unexplored safety problems. However, DMD is a multi-system disorder of obscure pathogenesis with some symptom complexes. It is desirable to have an insight into the molecular mechanisms of DMD in different organs/tissues and discover potential promising therapeutic targets.

Fibrosis has been increasingly accepted as the principal cause of muscle dysfunction in DMD (Desguerre et al., 2009), and it is an impediment for AVV-based gene delivery and stem cell delivery. Over the past few years, the molecular mechanisms underlying muscle fibrosis and the potential antifibrotic therapies have been studied. Most fibrosis-targeted therapeutic strategies exhibit promising results in pre-clinical experiments (Henderson et al., 2020). For example, the pro-fibrotic role of CTGF has been well-studied in several pathologies characterized by the development of fibrosis (Gonzalez and Brandan, 2019). Reduction of CTGF levels in mdx mice can significantly decrease fibrosis and improve skeletal muscle phenotype as well as functions. The CTGF-specific antibody, FG-3019, has shown promising anti-fibrosis effect in patients with idiopathic pulmonary fibrosis (IPF) and pancreatic cancer (Raghu et al., 2016). Furthermore, compared with the therapeutic strategies that target TGF-β, the therapeutic strategies targeting CTGF are considered safer since sustained systemic inhibition of TGF-β can result in side effects such as cardiac valve problems (Henderson et al., 2020). Besides, the TGF-β1 blockade can also induce carcinogenesis, owing to the role of TGF-β1 as an anti-proliferative mediator for most epithelial cell types (Henderson et al., 2020).

Most notably, even though some endpoints about muscle wasting (i.e., muscle mass) could be ameliorated with some of the tested drugs, most trials failed to show significant improvement in the defined clinical endpoints. More effective and safer potential targets for DMD are desired.

### Meta-Analysis for Identifying Potential Targets

Many gene variants are contained in the human genome, and some special gene variants can interact with the disease-causing mutation and further modulate the phenotype of DMD (Bello and Pegoraro, 2019). The bioinformatic analysis could be an effective tool for the identification of potential genetic targets. In this study, 133 DMD samples and 116 normal control samples from multiple microarray datasets were selected for the identification of DEGs. However, there is no gene that could be consistently identified as DEG in all 12 datasets. Possible reasons for this include: (i) The patient sample might be collected at different stages of disease progression. (ii) The number of genes was low in some datasets such as GSE32720 which only contains 3266 genes. (iii) Stringent quality control criteria were implanted to detect and discard low-quality genes. A meta-analysis that could combine information from multiple datasets and increase the reliability and generalizability identified a robust meta-gene profile comprising 121 DEGs. Hub genes identification and enrichment analysis on the DEGs demonstrated that ECM formation, immune response, bone development exerted significant roles in DMD pathogenesis. Furthermore, there are also several limitations in this meta-analysis. Firstly, the result of this meta-analysis might be also affected by the relatively small sample size of the included studies. Studies with a larger sample size and cross-validation in different populations and ethnic groups are warranted. Secondly, the present study utilized the public database to process secondary data mining and conduct bioinformatic analysis, which lacked clinical verification.

Despite these limitations, our meta-analysis still provides an overview of the involved biomarkers and DEGs in the pathogenesis of DMD for further experimental validation or consideration. Among 10 hub genes identified from the meta-analysis, seven genes, including FN1, CTGF, SPP1, POSTN, THBS2, COL4A2, and TGF-β1, are involved in ECM formation, implying anti-fibrotic therapy might serve as a necessary and important way to treat DMD, in addition to gene and cell therapies, in the future.

## Author Contributions

SY, ZC, GZ, ZZ, BZ, and YY made contributions to the concept design of the article, acquisition, and analysis of the review. SY and ZC drafted the manuscript. ZC covered the meta-analysis. NZ and HJ interpreted the data for the meta-analysis. All the authors reviewed the manuscript and approved the final version of the manuscript.

## Conflict of Interest

The authors declare that the research was conducted in the absence of any commercial or financial relationships that could be construed as a potential conflict of interest.

## Publisher’s Note

All claims expressed in this article are solely those of the authors and do not necessarily represent those of their affiliated organizations, or those of the publisher, the editors and the reviewers. Any product that may be evaluated in this article, or claim that may be made by its manufacturer, is not guaranteed or endorsed by the publisher.

## Footnotes

1
https://string-db.org/cgi/input.pl
